# The Effect Analysis of Strain Rate on Power Transmission Tower-Line System under Seismic Excitation

**DOI:** 10.1155/2014/314605

**Published:** 2014-06-29

**Authors:** Li Tian, Wenming Wang, Hui Qian

**Affiliations:** ^1^School of Civil and Hydraulic Engineering, Shandong University, Jinan 250061, China; ^2^Shandong Electric Power Engineering Consulting Institute Co., Ltd., Jinan 250013, China; ^3^School of Civil Engineering, Zhengzhou University, Zhengzhou 450001, China

## Abstract

The effect analysis of strain rate on power transmission tower-line system under seismic excitation is studied in this paper. A three-dimensional finite element model of a transmission tower-line system is created based on a real project. Using theoretical analysis and numerical simulation, incremental dynamic analysis of the power transmission tower-line system is conducted to investigate the effect of strain rate on the nonlinear responses of the transmission tower and line. The results show that the effect of strain rate on the transmission tower generally decreases the maximum top displacements, but it would increase the maximum base shear forces, and thus it is necessary to consider the effect of strain rate on the seismic analysis of the transmission tower. The effect of strain rate could be ignored for the seismic analysis of the conductors and ground lines, but the responses of the ground lines considering strain rate effect are larger than those of the conductors. The results could provide a reference for the seismic design of the transmission tower-line system.

## 1. Introduction

Power transmission tower-line system is an important component of a power system. Its reliable operation is of great significance in achieving the energy strategy of nationwide interconnection, power transmission from west to east, and mutual supply between north and south. So far, most of research attentions have been paid on the actions of static load, dynamic characteristic, and nonlinear time history analysis with only geometric nonlinear was taken into account [1–8]. However, several recent cases of damage to transmission towers during earthquakes have proved that the structure under strong earthquake excitation could enter nonlinear status. During the 1976 Tangshan earthquake, some transmission towers collapsed. In the 1999 CHI-CHI earthquake, a lot of transmission lines were broken and some towers collapsed [9]. In 2008, Sichuan electric network was damaged by the Wenchuan earthquake.

Therefore, studies about elastic-plastic analysis of transmission towers have been reported recently. Li et al. [10] calculated the dynamic characters of two different kinds of towers, and the two transmission towers were calculated with two different pieces of seismic records based on the simplified method of seismic calculation, and the nonlinear behaviors and plastic limit of transmission towers in different vibration directions subjected to earthquake action were summarized. Xiong et al. [11] investigated the elastic-plastic analysis of a long-span concrete filled steel-tube transmission tower under earthquake actions by the static and the dynamic method. The results showed a lot of elements enter the plastic status soon after earthquake action and there were some steel-tube elements that failed but no concrete filled steel-tube element failed at last times. Albermani et al. [12] presented a nonlinear analysis technique for transmission tower structures, and the proposed technique could be used to accurately predict structural failure, with the predictions confirmed by the results of an expensive full-scale test. It is necessary to consider the effect of strain rate on transmission tower-line system using elastic-plastic analysis method. However, there is no research about the analysis of strain rate effect on transmission tower-line system at present.

The experimental results have showed that the steel is a rate sensitive material, which has different mechanical properties under different strain rates. A few experimental studies have been carried out in recent years. ChangandLee [13] investigated the strain rate and strain rate history effects on the inelastic stress-strain behavior of annealed A-36 structural steel at room temperature under monotonic and cyclic loading conditions. Test results show relatively more significant strain rate sensitivity of the material for monotonic loading and less significant strain rate sensitivity for cyclic loading. Restrepo-Posada et al. [14] studied variables affecting cyclic behavior of reinforcing steel, and the effects of strain on the cyclic behavior of reinforcing steel can be very relevant when considering postearthquake retrofitting of reinforced concrete structures. Yang et al. [15] studied the dynamic tensile property of three steels, and the results showed that the yield strength and extension rate of the steel improved with strain rate increasing but the rate sensitivity was different. Song [16] carried out the dynamic cyclic loading test of steel, and the results showed that the yield strength of the steel improved with strain rate increasing, and the elastic modulus was not changed. At present general conclusion is that the yield strength and tensile strength of the steel have a certain improvement with strain rate increasing, and the increased range of yield strength is larger than the tensile strength, but the elastic modulus is not changed. The lower the strength of the steel is, the more obvious the effect of strain rate is. Strain rate has an influence on the response of the structure under earthquake excitation. It is unreasonable to calculate the seismic response of the structure using static analysis, and the effect of strain rate should be considered. It is very important to study the influence of strain rate on the nonlinear seismic response of the structure.

This paper aims to investigate the influence of the strain rate effect on the power transmission tower-line system under different ground motion intensities. A subroutine is developed in ABAQUS. The progressive collapse simulation method is proposed. Incremental nonlinear time history analysis method is adapted to calculate the response with and without considering the strain rate effect. The results could provide a reference for the seismic design of the transmission tower-line system.

## 2. Structural Model

**Figure 1:**
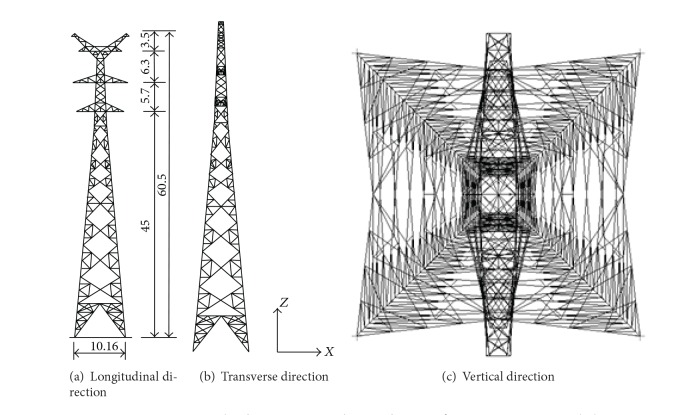
Longitudinal, transverse, and vertical views of a transmission tower (m).

The selected tower for the analysis is illustrated in Figure 1, which has a height of 60.5 m and a square base area of 10.16 m × 10.16 m at ground level. The angle steel with equal section is used for all tower members. Main members of the tower are made of Q345, and secondary members are made of Q235. The mechanical properties of Q345 and Q235 are shown in Table 1.

**Figure 2:**
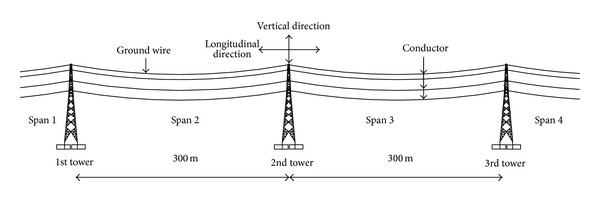
Three-dimensional finite element model of transmission tower-line system.

A transmission tower-line system consists of many towers and lines, and the coupling effects of tower and line are prominent. However, it is unrealistic to establish a model that includes all towers and lines. The numerical model contains three towers and four-span lines in the paper, which was verified to be reasonable [17, 18]. As shown in Figure 2, the transmission tower-line coupled system includes three towers (1st tower, 2nd tower, and 3rd tower) and four-span line (Span 1, Span 2, Span 3, and Span 4). The upper 8 cables are ground lines and the lower 24 cables are single bundled conductor. The spans of adjacent towers are all 300 m. The conductors and towers are connected with insulators and the materials for conductor and ground wire are steel-cored aluminum strand. The base points of the transmission tower are fixed on the ground, and the connections between transmission towers and lines are hinged. The side spans of the lines are hinged at the same height of the middle tower. The properties and performance indices of conductor, ground wire, and insulator are listed in Table 2.

The three-dimensional beam elements type, B31, with three translational and three rotational degrees of freedom per node, is employed to model the tower members, and the three-dimensional truss elements type, T3D2, with three translational degrees of freedom per node, is applied to model the lines and insulators in the ABAQUS software. Each tower contains 741 beam elements, each insulator contains one element, and the mesh selected for each conductor and ground wire consists of 100 truss elements.

## 3. Material Strain Rate and Analysis Method

### 3.1. Material Strain Rate

The strain rate of steel under seismic excitation is hard to exceed 1/*s*. The dynamic constitutive relationship model incorporated in the finite element analysis is as follows [19]:

(1) fyd =(1+cf lgε˙ε˙0 )fyscf=0.1709−3.289×10−4fys,

where $\dot{\varepsilon }$ is the current strain rate; ${\dot{\varepsilon }}_{0}$ is the quasistatic strain rate,  ${\dot{\varepsilon }}_{0}=2.5\;\times \;10^{-4}/s$; *f*_ys_ is the yield strength at quasistatic strain rate; *f*_yd_ is the dynamic yield strength at the current strain rate.

### 3.2. Theoretical Analysis

For the transmission tower-line system, the nonlinear dynamic analysis should be used because of its importance and complexity. The motion equation of structure under the seismic excitation is as follows:

(2) M(t)x¨(t)+C(t)x˙(t)+K(t)x(t)  =−M(t)(x¨u(t)+x¨v(t)+x¨w(t)),

where $\ddot{x}(t)$, $\dot{x}(t)$, and *x*(*t*) are the relative acceleration, velocity, and displacement vectors; ${\ddot{x}}_{u}(t)$, ${\ddot{x}}_{v}(t)$, and ${\ddot{x}}_{w}(t)$ are the ground motion accelerations in two horizontal and one vertical directions, respectively; *M*(*t*), *K*(*t*), and *C*(*t*) are the mass, stiffness, and damping matrices, respectively. Generally, *M*(*t*) is an invariant, while *K*(*t*) and *C*(*t*) change during the earthquake.

If the ground motion intensity is weak, the material cannot enter nonlinear status. The mass, stiffness, and damping matrices are irrelevant to strain rate. The strain rate has no influence on the elastic response analysis of the structure. If the ground motion intensity is strong, the material can enter nonlinear status. The stiffness matrix is relevant to strain rate, but the mass matrix is irrelevant to strain rate. The damping matrix is irrelevant to strain rate if the mass-damping is used. However, if stiffness-damping or Raleigh damping is chosen, the damping matrix is relevant to strain rate. Therefore, the strain rate has some influence on the plastic response analysis of the structure.

According to a series of ground motion intensity indices of monotone increasing of a specific seismic wave, the structure is analyzed using nonlinear time history analysis, and the nonlinear seismic response of the structure under different ground motion intensities can be obtained. The method is called incremental dynamic analysis (IDA) [20]. Multiple seismic waves are selected. The response of the structure is calculated by nonlinear time history analysis method with and without strain rate effect taken into account. The peak accelerations of the three-dimensional seismic waves are increased with equal proportions until progressive collapse of the structure occurs.

### 3.3. Progressive Collapse Simulation

**Figure 3:**
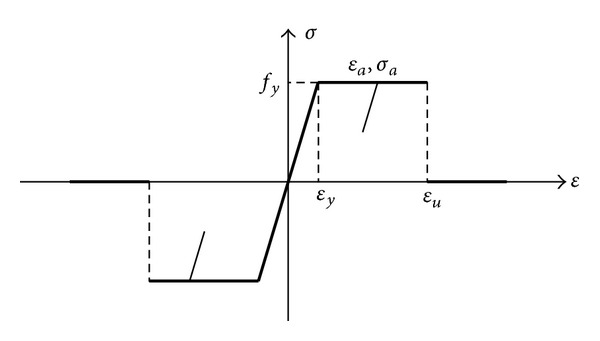
Stress-strain curve of steel.

Elastic-perfectly plastic material model is used, which is shown in Figure 3. The constitutive relationship is coded using the user subroutine VUMAT [21], which can be implemented in the advanced finite element program ABAQUS. According to the proposed method, once the strain exceeds ultimate strain, the stiffness of the element is zero while the mass remains the same.

For uniaxial loading, the stress-strain relationship of steel is as follows:

(3) $$\begin{array}{c} \sigma =\{\begin{array}{cc} E_{s}\varepsilon & \varepsilon \leq \varepsilon_{y},\\ f_{y} & \varepsilon_{y}\leq \varepsilon \leq \varepsilon_{u},\\ 0 & \varepsilon >\varepsilon_{u}, \end{array} \end{array}$$

where *ε* and *σ* are the strain and stress, respectively; *E*_*s*_ is the elastic modulus; *ε*_*y*_ and *f*_*y*_ are the yield strain and yield stress, respectively; *ε*_*u*_ is the ultimate strain.

For cyclic loading, the stress-strain relationship is as follows:

(4) $$\begin{array}{c} \sigma =\sigma_{a}+E_{s}\left(\varepsilon -\varepsilon_{a}\right), \end{array}$$

where *σ*_*a*_ and *ε*_*a*_ are the stress and strain of the starting point in the unloading curve, respectively.

## 4. The Selection of Seismic Waves 

Three seismic waves are selected: (1) Kobe wave (1995); (2) Northbridge wave (1994); (3) El Centro wave (1940). And the seismic waves include two horizontal components and one vertical component. The NS, WE, and vertical components of seismic wave coincide with the* Y*,* X*, and* Z* directions of the transmission tower-line system shown in Figure 2, respectively. The material of transmission tower under seismic excitation is hard to enter plastic status and is very hard to collapse. When the plastic analysis is carried out, the ground motion intensity requires very strong amplitude. The structure under very strong earthquake action is analyzed using elastic-plastic analysis method. The increasing of ground motion intensity is fast at the beginning of the incremental dynamic analysis. When the structure is close to collapse, the increasing of seismic intensity is slow, and the minimum increment of ground motion intensity is 1 m/s^2^.

## 5. Numerical Simulation and Discussion

The responses of the strain rate effect on the power transmission tower line system shown in Figure 2 under different ground motion intensities are investigated. The nonlinear time history analysis method is used in the paper. The geometric nonlinearity is taken into account due to large deformation of the transmission lines and the material nonlinearity of the system is considered. The strain rate effect on the maximum top displacement and base shear force of the transmission tower are studied. The strain rate effect on the maximum tension force and vertical displacement of the conductor and ground line are also investigated.

### 5.1. The Effect of Strain Rate on the Transmission Tower

The maximum top displacements of 2nd transmission tower under different seismic excitations are given in Table 3, and the table shows the responses of the transmission tower with and without considering the strain rate. It also can be seen from Table 3 that the transmission tower is easy to collapse under El Centro and Kobe earthquake actions, but it is hard to collapse under Northbridge earthquake action. The deformation capacity of the transmission tower under Kobe earthquake action is larger than those of the other two earthquake actions.

**Figure 4:**
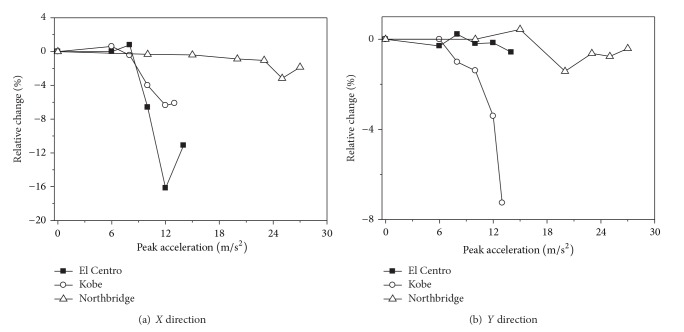
Relative changes of the maximum top displacements of 2nd transmission tower.

Figure 4 shows the relative changes of the maximum top displacements of 2nd transmission tower considering the effect of strain rate with the increasing ground motion intensity. It can be seen form Figure 4 that the effect of strain rate on the transmission tower may decrease or increase the maximum top displacements when the ground motion intensity is weak. When the transmission tower is close to collapse, the transmission tower considering the effect of strain rate significantly decreases the maximum top displacements. The effect of strain rate has little influence on the maximum top displacements of the transmission tower under Northbridge earthquake action. However, the effect of strain rate has a great influence on the maximum top displacements of the transmission tower under El Centro and Kobe earthquake actions, and the maximum reduction of the maximum top displacement is 16.2%. The effect of strain rate on the maximum top displacement of the transmission tower has an increasing tendency with the ground motion intensity increasing.

**Figure 5:**
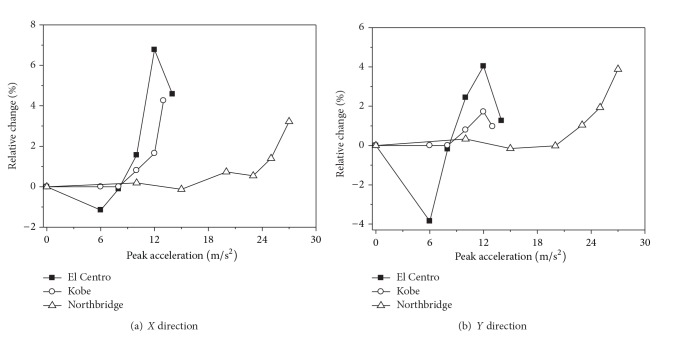
Relative changes of the maximum base shear forces of 2nd transmission tower.

The maximum base shear forces of 2nd transmission tower under different seismic wave excitations are shown in Table 4. It can be seen from Table 4 that the base shear forces of the transmission tower under Northbridge earthquake action are very large.  Figure 5 shows the relative changes of the maximum base shear forces of the transmission tower considering the effect of strain rate with the ground motion intensity increasing. The effect of strain rate on the transmission tower would decrease or increase the maximum base shear forces of the transmission tower. The nonlinear degree of the material is very low and the effect of strain rate is very small when the ground motion intensity is weak. The effect of strain rate would increase the maximum base shear forces of the transmission tower when the seismic ground motion is strong, and the maximum increase is 6.8%. The maximum base shear forces of the transmission tower considering strain rate effect are varied by less than 5%, but the effect of strain rate on the maximum base shear forces of the transmission tower is less than that of the maximum top displacements. The effect of strain rate on maximum base shear forces of the transmission tower has an increasing tendency with the ground motion increasing.

### 5.2. The Effect of Strain Rate on the Conductor and Ground Line

**Figure 6:**
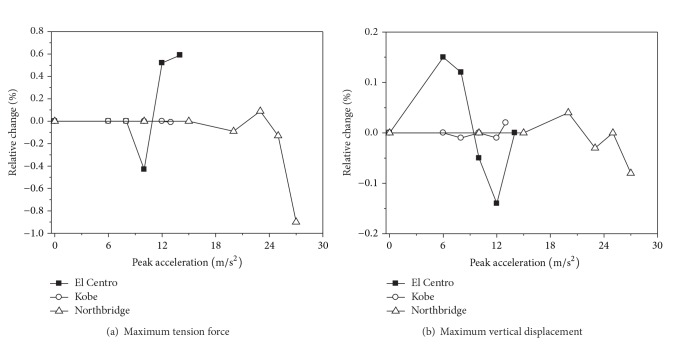
Relative changes of the conductor's maximum responses.

The maximum tension forces and vertical displacements of the conductor of span 2 are given in Table 5. Relative changes to the conductor's maximum response considering the effect of strain rate with the ground motion increasing are shown in Figure 6. It can be seen from the analysis results that the effect of strain rate has little influence on the response of the conductor, and the maximum responses of the conductor considering the effect of strain rate are varied by less than 1%; so the effect of strain rate on the conductor can be ignored. The material of the conductor cannot enter plastic status, and the effect of strain rate has no direct influence on the response of the conductor, but it could have an indirect influence on the conductor through the transmission tower.

**Figure 7:**
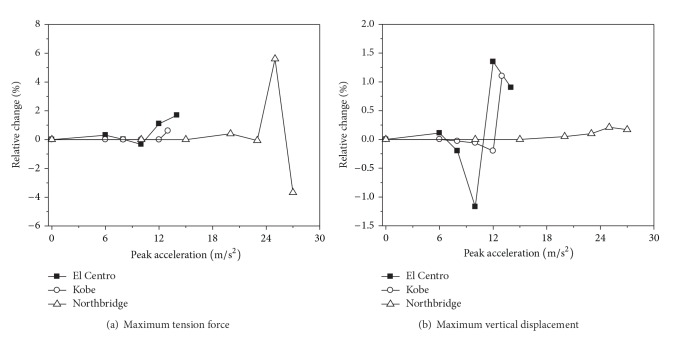
Relative changes of the ground wire's maximum responses.

Table 6 shows the maximum tension forces and vertical displacements of the ground line of span 2. It can be seen from Table 6 that the change of the response of the ground line is very little with the ground motion increasing. Relative changes of the ground line's maximum responses considering the effect of strain rate with the ground motion increasing are shown in Figure 7. The effect of strain rate has little influence on the ground line response, and the maximum responses of the ground line considering the effect of strain rate are varied by less than 6%. Comparing the response of the conductor and the ground line considering strain rate effect, the effect of strain rate on the ground line is more significant than that of the conductor. The reason is that the insulator length between the ground line and the transmission tower is shorter than that of between the conductor and the transmission tower.

## 6. Conclusion

The power transmission tower-line system is analyzed using incremental nonlinear time history analysis method, and the responses of strain rate on the transmission tower-line system are studied. Based on the numerical results, the following conclusions are drawn.

1. If the ground motion is weak, the effect of strain rate on the transmission tower would decrease the maximum top displacements in most of cases, but it would increase the maximum top displacements in few cases. The effect of strain rate on the transmission tower significantly decreases the maximum top displacements when the ground motion intensity of the transmission tower is close to collapse and the maximum reduction of the maximum top displacement is 16.2%.
2. If the ground motion is weak, the effect of strain rate on the transmission tower increases the maximum base shear forces in most of cases, but it would decrease the maximum base shear forces in few cases. The effect of strain rate on the transmission tower would increase the maximum base shear forces when the ground motion intensity of the transmission tower is close to collapse, and the maximum increase of the maximum base shear force is 6.8%.
3. The effects of strain rate on the response of the transmission tower-line system have an increasing tendency with the ground motion intensity increasing.
4. The effect of strain rate has little influence on the response of the conductor, and the maximum responses of the conductor considering strain rate are varied by less than 1%; so the effect of strain rate on the conductor can be ignored. Comparing the responses of the conductor and ground line considering the effect of strain rate, the effect of strain rate on the ground line is more significant than that of the conductor, but the maximum responses of the ground line considering strain rate effect are less than 6%.

This study demonstrates that the strain rate effect is very important to transmission tower-line system, especially for transmission tower. As many other type towers such as cat head type towers, cup towers, and guyed towers are also widely used in the powers transmission systems, more studies are deemed necessary to further investigate the strain rate effect on responses of these systems.

## Figures and Tables

**Table 1:** Mechanical properties of Q345 and Q235.

Material	Yield stress (Pa)	Young's modulus (Pa)	Poisson's ratio	Density (Kg/m^3^)
Q345	3.45*E* + 08	2.00*E* + 11	0.3	7800
Q235	2.35*E* + 08	2.00*E* + 11	0.3	7800

**Table 2:** Properties and performance indices of conductor, ground wire, and insulator.

Type	Area (m^2^)	Young's modulus (Pa)	Poisson's ratio	Density (Kg/m^3^)	Yield force (N)
Conductor	4.25*E* − 04	6.50*E* + 10	0.3	3172	98710
Ground wire	1.53*E* − 04	1.05*E* + 11	0.3	4631	74200
Insulator	0.02	7.65*E* + 10	0.2	7500	1000000

**Table 3:** Maximum top displacements of 2nd transmission tower.

Seismic wave	Peak acceleration (m/s^2^)	Displacement (m)			
		*X* direction		*Y* direction	
		Without considering strain rate	Considering strain rate	Without considering strain rate	Considering strain rate
El Centro	6.0	0.243	0.243	0.330	0.329
	8.0	0.256	0.258	0.453	0.454
	10.0	0.339	0.318	0.514	0.513
	12.0	0.460	0.396	0.618	0.617
	14.0	0.841	0.757	0.691	0.687
	15.0	Collapse	1.124	Collapse	0.706
	16.0	Collapse	Collapse	Collapse	Collapse

Kobe	6.0	0.498	0.501	0.505	0.505
	8.0	0.685	0.682	0.701	0.694
	10.0	1.012	0.973	0.949	0.936
	12.0	1.085	1.020	1.121	1.084
	13.0	1.177	1.109	1.241	1.157
	14.0	Collapse	1.196	Collapse	1.268
	15.0	Collapse	Collapse	Collapse	Collapse

Northbridge	10.0	0.306	0.305	0.329	0.329
	15.0	0.518	0.516	0.453	0.455
	20.0	0.573	0.568	0.499	0.492
	23.0	0.681	0.674	0.644	0.640
	25.0	0.748	0.725	0.664	0.659
	27.0	0.828	0.813	0.743	0.740
	29.0	Collapse	0.880	Collapse	0.752
	30.0	Collapse	Collapse	Collapse	Collapse

**Table 4:** Maximum base shear forces of 2nd transmission tower.

Seismic wave	Peak acceleration (m/s^2^)	Shear force (kN)			
		*X* direction		*Y* direction	
		Without considering strain rate	Considering strain rate	Without considering strain rate	Considering strain rate
El Centro	6.0	138.465	136.894	163.459	157.404
	8.0	140.797	140.648	214.633	214.248
	10.0	151.760	154.172	262.068	268.623
	12.0	166.223	178.293	303.575	316.353
	14.0	203.141	212.887	319.025	323.093
	15.0	Collapse	222.141	Collapse	307.916
	16.0	Collapse	Collapse	Collapse	Collapse

Kobe	6.0	198.513	198.516	228.912	228.911
	8.0	228.882	228.878	260.429	260.427
	10.0	279.233	281.514	283.561	285.820
	12.0	248.166	252.340	263.036	267.653
	13.0	279.085	291.489	256.623	259.124
	14.0	Collapse	301.570	Collapse	267.427
	15.0	Collapse	Collapse	Collapse	Collapse

Northbridge	10.0	247.764	248.244	216.227	216.941
	15.0	289.418	289.030	309.638	309.181
	20.0	393.213	396.100	453.791	453.690
	23.0	456.353	458.828	469.431	474.372
	25.0	467.501	474.122	464.471	473.617
	27.0	465.700	481.191	500.019	520.178
	29.0	Collapse	485.623	Collapse	523.600
	30.0	Collapse	Collapse	Collapse	Collapse

**Table 5:** Maximum responses of the conductor.

Seismic wave	Peak acceleration (m/s^2^)	Tension force (kN)		Vertical displacement (m)	
		Without considering strain rate	Considering strain rate	Without considering strain rate	Considering strain rate
El Centro	6.0	21.262	21.263	8.859	8.872
	8.0	21.244	21.245	8.625	8.635
	10.0	21.224	21.134	8.423	8.419
	12.0	22.089	22.205	8.420	8.408
	14.0	21.185	21.310	8.184	8.184
	15.0	Collapse	21.651	Collapse	8.197
	16.0	Collapse	Collapse	Collapse	Collapse

Kobe	6.0	21.321	21.321	8.629	8.629
	8.0	21.322	21.322	8.640	8.639
	10.0	21.322	21.323	8.674	8.674
	12.0	21.324	21.323	8.707	8.706
	13.0	21.361	21.359	8.756	8.758
	14.0	Collapse	21.366	Collapse	8.790
	15.0	Collapse	Collapse	Collapse	Collapse

Northbridge	10.0	21.323	21.323	8.377	8.377
	15.0	21.324	21.324	8.081	8.081
	20.0	22.239	22.219	7.386	7.389
	23.0	21.326	21.346	7.219	7.217
	25.0	21.545	21.517	7.220	7.220
	27.0	21.617	21.424	7.221	7.215
	29.0	Collapse	22.203	Collapse	7.223
	30.0	Collapse	Collapse	Collapse	Collapse

**Table 6:** Maximum responses of the ground line.

Seismic wave	Peak acceleration (m/s^2^)	Tension force (kN)		Vertical displacement (m)	
		Without considering strain rate	Considering strain rate	Without considering strain rate	Considering strain rate
El Centro	6.0	17.239	17.292	3.509	3.513
	8.0	17.847	17.851	3.501	3.494
	10.0	18.651	18.589	4.428	4.377
	12.0	19.282	19.496	4.546	4.608
	14.0	19.183	19.512	4.276	4.315
	15.0	Collapse	19.965	Collapse	4.716
	16.0	Collapse	Collapse	Collapse	Collapse

Kobe	6.0	17.292	17.292	3.484	3.484
	8.0	17.292	17.291	3.485	3.484
	10.0	17.291	17.289	3.614	3.612
	12.0	17.292	17.290	3.586	3.579
	13.0	17.305	17.412	3.968	4.012
	14.0	Collapse	17.460	Collapse	4.163
	15.0	Collapse	Collapse	Collapse	Collapse

Northbridge	10.0	17.296	17.296	5.358	5.358
	15.0	22.424	22.423	5.487	5.487
	20.0	17.539	17.611	5.739	5.742
	23.0	20.882	20.870	5.253	5.258
	25.0	20.115	21.310	5.623	5.635
	27.0	20.790	20.053	5.152	5.161
	29.0	Collapse	21.136	Collapse	7.519
	30.0	Collapse	Collapse	Collapse	Collapse
